# Comparison of the SureX^®^ HPV genotyping test with the Digene Hybrid Capture^®^ 2 test in cervical cancer screening

**DOI:** 10.3389/fonc.2025.1627935

**Published:** 2025-07-24

**Authors:** Hehao Sun, Hong Wang, Yin Liu, Huifang Xu, Peipei Chen, Xingai Sun, Mengjie Li, Peiyao Li, Kunyao Li, Liyang Zheng, Shaokai Zhang

**Affiliations:** Department of Cancer Epidemiology and Prevention, Affiliated Cancer Hospital of Zhengzhou University/Henan Cancer Hospital, Henan Engineering Research Center of Cancer Prevention and Control, Henan International Joint Laboratory of Cancer Prevention, Zhengzhou, China

**Keywords:** human papillomavirus, SureX ^®^ HPV test, HC2 test, genotypes, cervical cancer screening

## Abstract

**Introduction:**

Cervical cancer, predominantly caused by high-risk human papillomavirus (HR-HPV) infections, is a critical public health issue. Effective screening is essential. This study compares the SureX^®^ HPV genotyping test, which detects 25 HPV types, with the widely used Digene Hybrid Capture^®^ 2 (HC2) test, which targets 13 HR-HPV types, to determine their effectiveness in cervical cancer screening among Chinese women.

**Methods:**

From September to December 2016, women aged 21–64 years in Shanxi Province were screened for cervical cancer using both SureX^®^ HPV and HC2 tests. Women with abnormal cytology test would be referred for colposcopy and biopsied if necessary. Concordance rates and kappa coefficients were calculated to analyze the concordance between the two tests. Sensitivity and specificity for detecting cervical intraepithelial neoplasia grade 2 and higher (CIN2+) cases of the two tests were calculated.

**Results:**

Among 3028 subjects analyzed in this study, the positive rate of the common set of 13 HR-HPV types for the SureX^®^ HPV test was 15.0%, slightly higher than HC2 test’s 13.5%. The overall concordance rate was 93.9% (95% CI: 92.95%-94.66%), with a kappa coefficient of 0.749 (0.715-0.783). Both tests demonstrated a sensitivity of 80.00% (54.81%-92.95%) for detecting CIN2+, with specificities of 85.30% (83.99%-86.52%) for SureX^®^ HPV and 86.82% (85.57%-87.98%) for HC2. For CIN3+ detection, both tests had 100% (67.56%-100%) sensitivity, with specificities of 85.20% (83.89%-86.42%) for SureX^®^ HPV and 86.72% (85.46%-87.89%) for HC2.

**Discussion:**

The SureX^®^ HPV test exhibited excellent concordance with HC2 in detecting the common 13 HR-HPV types and similar sensitivity for identifying CIN2+ cases. Its broader capability to detect 25 HPV genotypes positions it as a promising option for cervical cancer screening.

## 1 Introduction

Cervical cancer is a major public health problem. With an estimated 661,021 new cases and 348,189 deaths in 2022 worldwide, this disease ranks as the fourth most frequently diagnosed cancer and the fourth cause of cancer deaths among women (1). Experimental as well as epidemiological studies have identified that more than 90% of cervical cancers are caused by infection with human papillomavirus (HPV) (2–5). At present, more than 450 HPV genotypes have been identified including 13 high-risk human papillomavirus (HR-HPV), which are responsible for cervical neoplasia and other anogenital and oropharyngeal cancers (6, 7).

Cytology-based and HPV-based cervical cancer screening, colposcopy and histological diagnosis followed by the treatment of cervical intraepithelial neoplasms (CINs) have been widely used worldwide and reduced population-level cervical cancer incidence and mortality (4, 8–10). Compared with cytology-based test, HPV-based test demonstrated better sensitivity against the detection of cervical cancer and precursors, and could extend the screening interval (11, 12). In 2021, the World Health Organization (WHO) recommended the use of DNA-based HPV testing as a first-choice screening method for cervical cancer (13).

Up to date, there are a variety of HR-HPV testing assays. The digene Hybrid Capture^®^ 2 (HC2) HR-HPV DNA Test is the first HR-HPV testing assay that was approved by the U.S. Food and Drug Administration (FDA) for cervical cancer screening and management. It’s an *in vitro* nucleic acid hybridization assay designed for the qualitative detection of 13 HR-HPV types in cervical specimens. HC2 has been extensively validated and remains widely used in clinical practice due to its robust performance. However, it does not provide individual genotyping information, particularly for HPV 16 and 18, which are known to confer the highest risk of cervical carcinogenesis. Without specific genotype information, risk-based triage becomes less accurate, potentially leading to over-referral, unnecessary colposcopies, and increased patient anxiety. As genotype-specific management has become increasingly emphasized in screening guidelines (14, 15), assays capable of identifying individual HPV types may offer additional clinical value in risk stratification and patient management.

The SureX^®^ HPV genotyping test (SureX^®^ HPV test) is a novel HPV DNA detection method to detect and genotype 25 HPV types (HPV 6, 11, 16, 18, 26, 31, 33, 35, 39, 42, 43, 44, 45, 51, 52, 53, 56, 58, 59, 66, 68, 73, 81, 82 and 83). In this study, we evaluated the performance of the SureX^®^ HPV test in comparison with the HC2 test for cervical cancer screening among Chinese women, focusing on the detection of a common set of 13 HR-HPV types (HPV 16, 18, 31, 33, 35, 39, 45, 51, 52, 56, 58, 59, and 68). The results may provide evidence for the clinical applicability of the SureX^®^ HPV test as an alternative screening tool, offering comparable performance to HC2 while enabling broader genotype detection.

## 2 Methods

### 2.1 Study population

From September 2016 to December 2016, eligible women were recruited for cervical cancer screening program in Yangcheng County maternal and child health hospital, Shanxi, China. Women would be included if they: (1) were aged 21–64 years old with intact cervix; (2) had a quite well health condition and an expected good adherence to accept routine screening (including colposcopy and biopsy) for cervical cancer; (3) gave a signed informed consent. Women would be excluded if they: (1) were pregnant or within 8 weeks postpartum; (2) had a history of cervical surgery or pelvic radiotherapy; (3) had a history of cervical cancer or precancerous lesions. This study was approved by the Institutional Review Board of Affiliated Cancer hospital of Zhengzhou University.

### 2.2 Screening procedures

**Figure 1:**
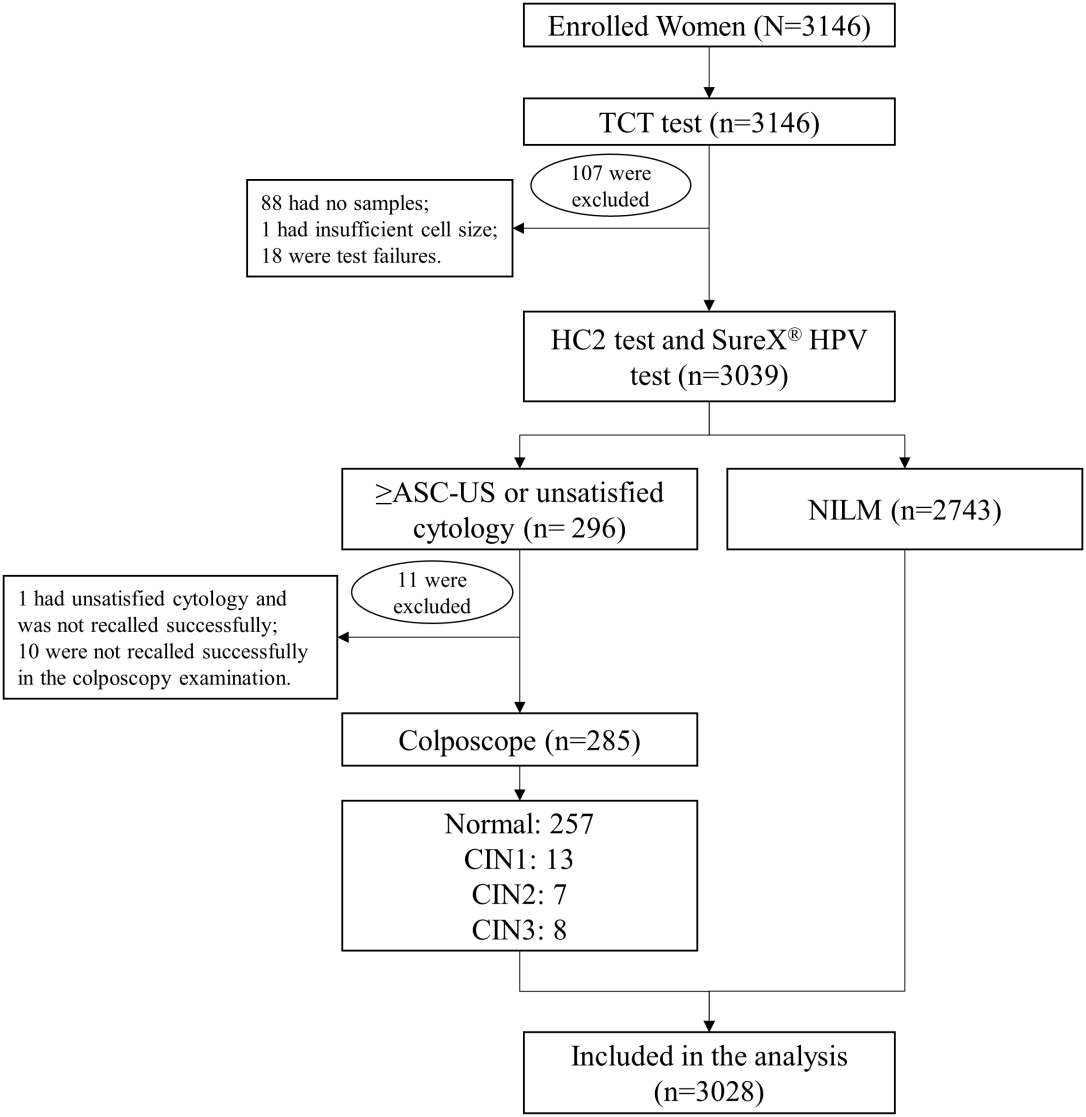
Flowchart of the cervical cancer screening procedure. Abbreviations: TCT, Thinprep liquid-based cytologic test; ASC-US, atypical squamous cells undetermined significance; NILM, negative for intraepithelial lesion or malignancy; CIN, cervical intraepithelial neoplasia.

Eligible women were enrolled after they signed informed consent. The gynecological examination was conducted by a physician, and two samples of cervical exfoliated cells were collected for cytology test and HPV test by HC2 test. To evaluate the performance of SureX^®^ HPV test, the remaining samples of cervical swab specimens previously examined by HC2 test were retrieved and subjected to SureX^®^ HPV test. Women with abnormal cytology test would be referred for colposcopy. The histological result obtained after colposcopy was regarded as the final diagnosis (**Figure 1**).

### 2.3 Cytology test

The Thinprep liquid-based cytologic test (TCT) was adopted to detect the cytology. All cytological samples were evaluated according to the Bethesda system (TBS) with following results: negative for intraepithelial lesion or malignancy (NILM), atypical squamous cells undetermined significance (ASC-US), atypical squamous cells‐cannot exclude HSIL (ASC-H), low-grade squamous intraepithelial lesion (LSIL), high-grade squamous intraepithelial lesion (HSIL), squamous of cervical carcinoma (SCC), atypical glandular cells (AGC).

### 2.4 HC2 test

The HC2 test (Qiagen, Gaithersburg, MD, USA) is a sandwich capture molecular hybridization assay, combining chemiluminescence-based signal amplification with nucleic acid hybridization principles. It specifically detects 13 HR-HPV types HPV (16, 18, 31, 33, 35, 39, 45, 51, 52, 56, 58, 59 and 68). The process involves the capture of RNA-DNA hybrids on an antibody-coated microtiter plate surface. Following immobilization, these hybrids are detected by introducing an alkaline phosphatase-conjugated antibody to the RNA-DNA complexes. Subsequent addition of a chemiluminescent substrate, cleaved by alkaline phosphatase, generates luminescence. A luminometer is employed to semiquantitatively measure the emitted light in relative light units (RLU). Samples registering measurements below the 1.0 RLU cut-off are interpreted as negative.

### 2.5 SureX^®^ HPV test

The SureX^®^ HPV test (Ningbo HEALTH Gene Technologies Co., Ltd., China) uses multiplex polymerase chain reaction (PCR) and capillary electrophoresis to amplify target HPV DNA. A total of 25 HPV types (HPV 6, 11, 16, 18, 26, 31, 33, 35, 39, 42, 43, 44, 45, 51, 52, 53, 56, 58, 59, 66, 68, 73, 81, 82 and 83) were detected and genotyped. According to the length of specific amplified fragments, DNA fragments of oncogenes E6/E7 of 25 HPV types were cloned, PCR-amplified, and recovered via gel electrophoresis in the laboratory. In addition, the concentration of each DNA fragment was measured by Agilent 2100, and the copy number was calculated. Finally, each DNA fragment was mixed to obtain a high concentration HPV positive control (10,000,000 copies/μL). Therefore, through PCR amplification of the target DNA based on the length of the PCR product, the SureX^®^ HPV test achieves accurate and comprehensive genotyping results.

### 2.6 Colposcopy and histological diagnosis

Women with abnormal cytology test (≥ASC-US or unsatisfied cytology test) would be referred for colposcopy. Based on colposcopy results, samples may be taken for histopathological examination if necessary. The histopathological diagnosis was classified according to the WHO histological criteria for cervical tumors and was used as the gold standard, with cervical intraepithelial neoplasia grade 2 and higher (CIN2+) considered positive.

### 2.7 Statistical analysis

All statistical analyses were conducted using SAS 9.4 and OpenEpi (available at http://www.openepi.com/). Concordance rates and kappa coefficients with 95% confidence intervals (CIs) were calculated to evaluate the agreement between HC2 test and SureX^®^ HPV test for detecting the 13 common HR-HPV types. Taking colposcopy and pathological diagnosis as the gold standard, the sensitivity, specificity, positive predictive value, negative predictive value of HC2 test and SureX^®^ HPV test in detecting CIN2 + and CIN3 + lesions were calculated using the “Screening” module in OpenEpi, with the Wilson Score method applied to derive the 95% CIs. McNemar’s test was used to compare paired proportions between the two HPV tests. All differences with P values of <0.05 (two-tailed) were considered statistically significant.

## 3 Results

### 3.1 Characteristics of the study population

A total of 3146 women were enrolled in the cervical cancer screening program. In this study, a total of 118 subjects were excluded, of which 88 were “no samples”, 1 was “insufficient sample volume”, 18 were “test failures”, 1 had unsatisfied cytology and was not recalled successfully, and 10 were not recalled successfully in the colposcopy examination. Finally, data of 3028 subjects were analyzed in this study, with the mean age of 45.5 ± 7.83 years old.

### 3.2 Comparison of positive rate for SureX^®^ HPV test and HC2 test

Of all the 3028 women, 2743 (90.6%) were diagnosed as NILM, 285 (9.4%) were ASC‐US or worse. The overall positive rates of the common set of 13 HR-HPV types for the SureX^®^ HPV test and HC2 test were 15.0% and 13.5%, respectively. In total, a greater percentage of specimens were positive using the SureX^®^ HPV test (P<0.05) (**Table 1**).

**Table 1:** Results of the SureX^®^ HPV and HC2 test by cytological and histopathological category.

Category	Total (%)	SureX^®^ HPV test (%)		HC2 test (%)	
		Positive	Negative	Positive	Negative
Cytology					
NILM	2743 (90.6)	395 (14.4)	2348 (85.6)	348 (12.7)	2395 (87.3)
ASC-US	207 (6.8)	44 (21.3)	163 (78.7)	43 (20.8)	164 (79.2)
LSIL	75 (2.5)	13 (17.3)	62 (82.7)	15 (20.0)	60 (80.0)
HSIL	3 (0.1)	3 (100)	0 (0)	3 (100)	0 (0)
Histology					
Normal	3000 (99.1)	437 (14.6)	2563 (85.4)	391 (13.0)	2609 (87.0)
CIN1	13 (0.4)	6 (46.2)	7 (53.8)	6 (46.2)	7 (53.8)
CIN2	7 (0.2)	4 (57.1)	3 (42.9)	4 (57.1)	3 (42.9)
CIN3	8 (0.3)	8 (100)	0 (0)	8 (100)	0 (0)
Total	3028 (100)	455 (15.0)	2573 (85.0)	409 (13.5)	2619 (86.5)

NILM, negative for intraepithelial lesion or malignancy; ASC-US, atypical squamous cells of undetermined significance; LSIL, low-grade squamous intraepithelial lesion; HSIL, high-grade squamous intraepithelial lesion; CIN: cervical intraepithelial neoplasia.

The positive rate of the SureX^®^ HPV test and HC2 test were completely consistent in women with pathological diagnoses of CIN1, CIN2 and CIN3. However, in women with normal pathological diagnoses, the HPV positivity rate of SureX^®^ HPV test is higher than that of HC2 test. More details can be found in **Table 1**.

**Figure 2:**
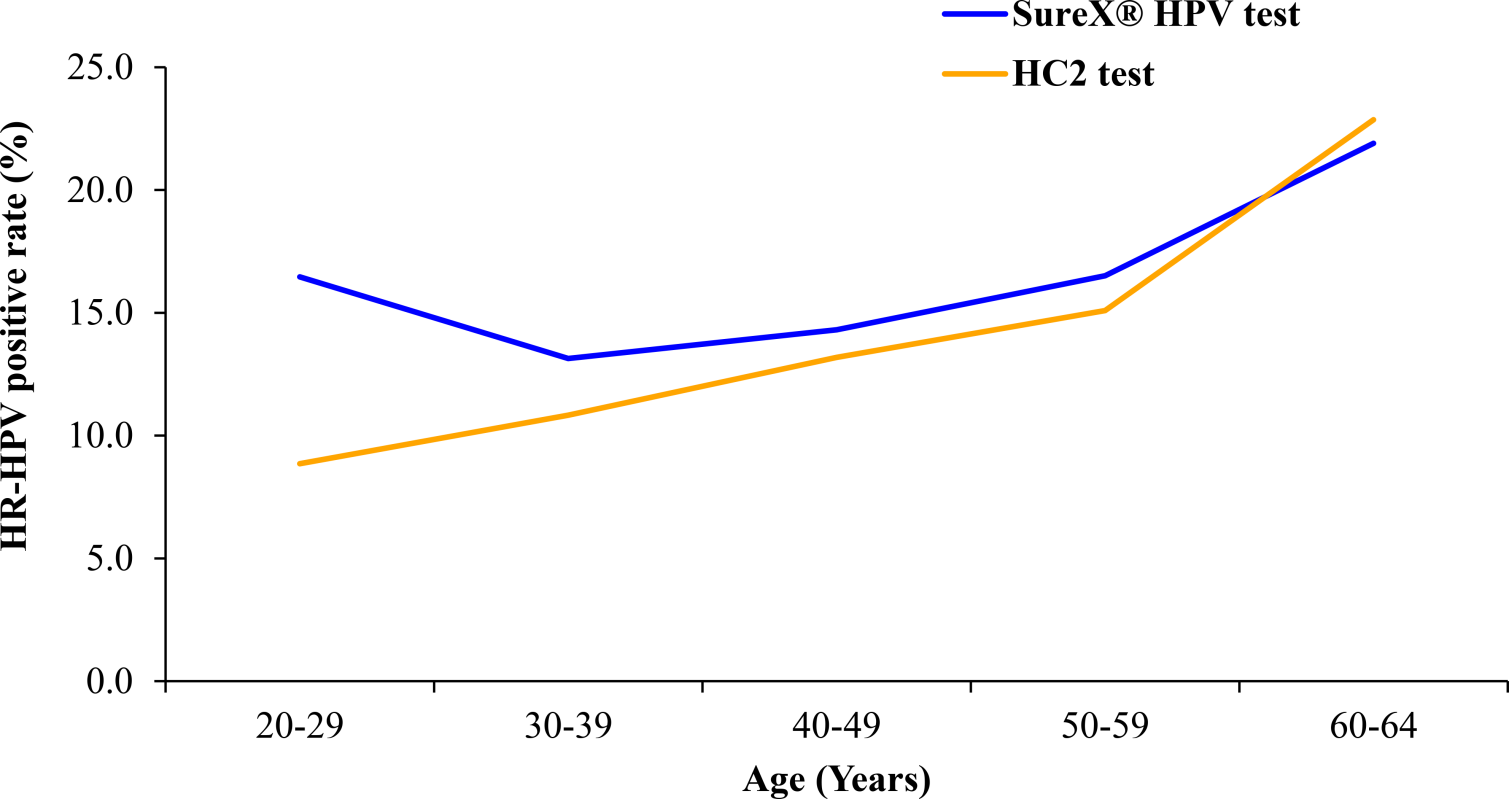
HR-HPV positive rates for SureX^®^ HPV and HC2 test in different age groups.

The HR-HPV prevalence for both the SureX^®^ HPV test and HC2 test was assessed across different age groups, as illustrated in **Figure 2**. The prevalence of HR-HPV exhibited an upward trend corresponding to the increasing age of the women in the study. Notably, in the 20–29 age group, the HR-HPV positive rate was higher for the SureX^®^ HPV test compared to the HC2 test.

Furthermore, the SureX^®^ HPV test provided detection of 25 HPV genotypes in total, with an overall HPV positivity rate of 22.0%. The genotype-specific positivity rates for all 25 types are summarized in **Supplementary Table 1**.

### 3.3 Concordance between the SureX^®^ HPV test and the HC2 test

The **Table 2** presents the overall concordance rate and age-specific rates for the SureX^®^ HPV test and HC2 test. The overall concordance rate was 93.9% (95% CI: 92.95%-94.66%), with a kappa coefficient of 0.749 (95% CI: 0.715-0.783). Variations in concordance rates and kappa coefficients were observed across different age groups, with the most optimal performance observed in the 60–64 age group.

**Table 2:** Concordance between the SureX^®^ HPV test and the HC2 test, overall and by age groups.

SureX^®^ HPV test	HC2 test		Concordance rate (%, 95% CI)	Kappa coefficient (95% CI)
	Positive	Negative		
Overall			93.9 (92.95, 94.66)	0.749 (0.715, 0.783)
Positive	339	116		
Negative	70	2503		
Age group				
20–29 years			92.4 (84.4, 96.47)	0.661 (0.416, 0.906)
Positive	7	6		
Negative	0	66		
30–39 years			94.9 (92.7, 96.39)	0.756 (0.672, 0.841)
Positive	53	21		
Negative	8	481		
40–49 years			93.6 (92.19, 94.74)	0.729 (0.677, 0.782)
Positive	151	54		
Negative	38	1190		
50–59 years			93.6 (91.78, 95.09)	0.761 (0.700, 0.822)
Positive	107	33		
Negative	21	687		
60–64 years			95.2 (89.33, 97.95)	0.863 (0.746, 0.980)
Positive	21	2		
Negative	3	79		

CI, confidence interval.

### 3.4 The sensitivity and specificity of SureX^®^ HPV test and HC2 test for the detection of CIN2+ and CIN3+ cases

For the detection of CIN2+, the sensitivity of both SureX^®^ HPV test and HC2 test was 80.00% (95% CI: 54.81%-92.95%); the specificity of SureX^®^ HPV test and HC2 test were 85.30% (95% CI, 83.99%-86.52%) and 86.82% (95% CI, 85.57%-87.98%), respectively. Regarding to the detection of CIN3+, the sensitivity of both tests was 100% (95% CI: 67.56%-100%); the specificity of SureX^®^ HPV test and HC2 test were 85.20% (95% CI, 83.89%-86.42%) and 86.72% (95% CI, 85.46%-87.89%), respectively (**Table 3**).

**Table 3:** The sensitivity and specificity of SureX^®^ HPV test and HC2 test for the detection of CIN2+ cases.

HPV testing	Cases	Non-cases	Sensitivity	Specificity	PPV	NPV
			(%, 95%CI)	(%, 95%CI)	(%, 95%CI)	(%, 95%CI)
CIN2+						
SureX^®^ HPV test						
Positive	12	443	80.00	85.30	2.64	99.88
Negative	3	2570	(54.81, 92.95)	(83.99, 86.52)	(1.52, 4.55)	(99.66, 99.96)
HC2 test						
Positive	12	397	80.00	86.82	2.93	99.89
Negative	3	2616	(54.81, 92.95)	(85.57, 87.98)	(1.69, 5.06)	(99.66, 99.96)
CIN3+						
SureX^®^ HPV test						
Positive	8	447	100	85.20	1.76	100
Negative	0	2573	(67.56, 100)	(83.89, 86.42)	(0.89, 3.43)	(99.85, 100)
HC2 test						
Positive	8	401	100	86.72	1.96	100
Negative	0	2619	(67.56, 100)	(85.46, 87.89)	(0.99, 3.81)	(99.85, 100)

HPV, Human Papillomavirus; CI, confidence interval; PPV, positive predictive value; NPV, negative predictive value; CIN, cervical intraepithelial neoplasia.

## 4 Discussion

The DNA-based HPV testing has been recommended by WHO for a first-choice screening method for cervical cancer and been widely used worldwide (13). The HC2 test, approved by FDA, was the pioneering HR-HPV screening method designated to qualitatively detect 13 specific HR-HPV types (HPV 16, 18, 31, 33, 35, 39, 45, 51, 52, 56, 58, 59 and 68). Despite its initial prominence, the limitations of the HC2 test hinder its application in cervical cancer screening in China. Addressing this, the SureX^®^ HPV test has been introduced as a novel method capable of detecting and genotyping 25 HPV types. In response to the limitations posed by existing technologies, the SureX^®^ HPV test introduces a distinctive approach by utilizing a multiplex PCR-capillary electrophoresis method. This innovative technique enables the simultaneous detection and identification of 25 HPV genotypes in a single tube, with a specific focus on the oncogenic E6/E7 regions. With the aim of enhancing screening efficiency, the SureX^®^ HPV test emerges as a promising alternative to established methods. In this study, we compared the performance of the SureX^®^ HPV test and the HC2 test in screening cervical cancer among Chinese women, with a specific focus on detecting the common set of 13 HR-HPV types. The results revealed an outstanding concordance rate between the SureX^®^ HPV test and the HC2 test.

The overall agreement rate was found to be 93.9% between the SureX^®^ HPV and the HC2 test. Notably, the robust concordance rates were observed across all age groups, affirming the reliability of the SureX^®^ HPV test in comparison to the HC2 test for detecting HR-HPV types. In addition, the overall agreement demonstrated comparability with values reported in other studies that compared HC2 with various PCR-based methods (16, 17). While our findings align with the results of several published studies comparing HC2 with other HR-HPV screening tests (18, 19), it is essential to acknowledge a potential underestimation of agreement between the two tests. This discrepancy may arise from the alkali condition of denatured HC2 samples, leading to DNA degradation over time. Consequently, this could reduce the level of remaining HR-HPV DNA available for detection by the SureX^®^ HPV test. In addition to the comparative analysis, our study identified a correlation between HR-HPV prevalence and the age of the women involved. The prevalence of HR-HPV in our study population exhibited an upward trend with increasing age, a pattern consistent with previous research findings (20, 21).

The method of DNA-based HPV test for the screening of cervical cancer and precancerous lesions was characterized by high sensitivity, while specificity being closely associated with the positive rate of HPV (22, 23). The specificity differed greatly among different populations. In the study, whether considering CIN2+ or CIN3+, both the SureX^®^ HPV test and HC2 test exhibited the same sensitivity, and their specificity values were approximately equal. Notably, certain studies have reported that the specificity of HPV DNA detection can fall below 50% (24, 25). Furthermore, following an extensive analysis of clinical samples, the HC2 test has emerged as the “gold standard” for HR-HPV detection (26–28). Consequently, there exists a reasonable consistency between the results obtained from the SureX^®^ HPV test and those from the HC2 test.

Furthermore, in contrast to previously developed and widely used HPV detection methods, such as real-time PCR, the SureX^®^ HPV test, utilizing a multiplex PCR-capillary electrophoresis method, demonstrates the capability to detect and identify 25 HPV genotypes in a single tube, with a focus on specific oncogenic E6/E7. The efficiency is notable, as approximately 96 specimens can be detected and reported within approximately 5 hours when utilizing a 24-channel-equipped capillary electrophoresis platform.

In summary, our study compared a novel HPV genotyping test, the SureX^®^ HPV test, with the HC2 test, revealing a robust consistency in HR-HPV detection. And the sensitivity and specificity for CIN2+ lesions and CIN3+ lesions of the SureX^®^ HPV test were equivalent to that of the HC2 test. Therefore, SureX^®^ HPV test, as a novel method for detecting and genotyping 25 HPV types in a single analysis, is an accurate, safe, and cost-effective HPV detection method, which may become a feasible alternative to HC2 test in cervical cancer screening.

## Data Availability

The datasets presented in this article are not readily available because individual-level study data cannot be shared publicly due to its confidentiality. Aggregated data, from which participants are not identifiable, can be shared upon request. Requests to access the datasets should be directed to Shaokai Zhang, shaokaizhang@126.com

## References

[1] Bray F Laversanne M Sung H Ferlay J Siegel RL Soerjomataram I. Global cancer statistics 2022: GLOBOCAN estimates of incidence and mortality worldwide for 36 cancers in 185 countries. CA: Cancer J Clin. (2024) 74:229–63. doi: 10.3322/caac.21834, PMID: 38572751

[2] Xu M Cao C Wu P Huang X Ma D. Advances in cervical cancer: current insights and future directions. Cancer Commun (London England). (2025) 45:77–109. doi: 10.1002/cac2.12629, PMID: 39611440 PMC11833674

[3] Olusola P Banerjee HN Philley JV Dasgupta S. Human papilloma virus-associated cervical cancer and health disparities. Cells. (2019) 8:622. doi: 10.3390/cells8060622, PMID: 31234354 PMC6628030

[4] Okunade KS. Human papillomavirus and cervical cancer. J obstetrics gynaecology: J Institute Obstetrics Gynaecology. (2020) 40:602–8. doi: 10.1080/01443615.2019.1634030, PMID: 31500479 PMC7062568

[5] Zhang S Xu H Zhang L Qiao Y. Cervical cancer: Epidemiology, risk factors and screening. Chin J Cancer Res = Chung-kuo yen cheng yen chiu. (2020) 32:720–8. doi: 10.21147/j.issn.1000-9604.2020.06.05, PMID: 33446995 PMC7797226

[6] Burd EM. Human papillomavirus laboratory testing: the changing paradigm. Clin Microbiol Rev. (2016) 29:291–319. doi: 10.1128/CMR.00013-15, PMID: 26912568 PMC4786885

[7] Crosbie EJ Einstein MH Franceschi S Kitchener HC. Human papillomavirus and cervical cancer. Lancet (London England). (2013) 382:889–99. doi: 10.1016/S0140-6736(13)60022-7, PMID: 23618600

[8] Kaufman HW Alagia DP Chen Z Onisko A Austin RM. Contributions of liquid-based (Papanicolaou) cytology and human papillomavirus testing in cotesting for detection of cervical cancer and precancer in the United States. Am J Clin pathology. (2020) 154:510–6. doi: 10.1093/ajcp/aqaa074, PMID: 32637991 PMC7523581

[9] Learman LA Garcia FAR. Screening for cervical cancer: new tools and new opportunities. Jama. (2018) 320:647–9. doi: 10.1001/jama.2018.11004, PMID: 30140859

[10] Perkins RB Wentzensen N Guido RS Schiffman M. Cervical cancer screening: A review. Jama. (2023) 330:547–58. doi: 10.1001/jama.2023.13174, PMID: 37552298

[11] Fontham ETH Wolf AMD Church TR Etzioni R Flowers CR Herzig A. Cervical cancer screening for individuals at average risk: 2020 guideline update from the American Cancer Society. CA: Cancer J Clin. (2020) 70:321–46. doi: 10.3322/caac.21628, PMID: 32729638

[12] Koliopoulos G Nyaga VN Santesso N Bryant A Martin-Hirsch PP Mustafa RA. Cytology versus HPV testing for cervical cancer screening in the general population. Cochrane Database Syst Rev. (2017) 8:CD008587. doi: 10.1002/14651858.CD008587.pub2, PMID: 28796882 PMC6483676

[13] WHO. WHO guideline for screening and treatment of cervical pre−cancer lesions for cervical cancer prevention[EB/OL] (2021). Available online at: https://www.who.int/publications-detail-redirect/9789240030824 (Accessed April 17, 2025). 34314129

[14] Perkins RB Guido RS Castle PE Chelmow D Einstein MH Garcia F. ASCCP risk-based management consensus guidelines for abnormal cervical cancer screening tests and cancer precursors. J lower genital tract Dis. (2019) 24:102–31. doi: 10.1097/lgt.0000000000000525, PMID: 32243307 PMC7147428

[15] Huh WK Ault KA Chelmow D Davey DD Goulart RA Garcia FA. Use of primary high-risk human papillomavirus testing for cervical cancer screening: interim clinical guidance. Gynecologic Oncol. (2015) 136:178–82. doi: 10.1016/j.ygyno.2014.12.022, PMID: 25579107

[16] Cook DA Smith LW Law J Mei W van Niekerk DJ Ceballos K. Aptima HPV Assay versus Hybrid Capture(^®^) 2 HPV test for primary cervical cancer screening in the HPV FOCAL trial. J Clin virology: Off Publ Pan Am Soc Clin Virology. (2017) 87:23–9. doi: 10.1016/j.jcv.2016.12.004, PMID: 27988420

[17] Huijsmans CJ Geurts-Giele WR Leeijen C Hazenberg HL van Beek J de Wild C. HPV Prevalence in the Dutch cervical cancer screening population (DuSC study): HPV testing using automated HC2, cobas and Aptima workflows. BMC cancer. (2016) 16:922. doi: 10.1186/s12885-016-2961-2, PMID: 27894291 PMC5127037

[18] Rao A Sandri MT Sideri M Young S Sharma A Behrens C. Comparison of hybrid capture 2 High-Risk HPV results in the low positive range with cobas^®^ HPV Test results from the ATHENA study. J Clin virology: Off Publ Pan Am Soc Clin Virology. (2013) 58:161–7. doi: 10.1016/j.jcv.2013.06.041, PMID: 23895930

[19] Zhao G Tian Y Du Y Sun J Wang Z Ma Y. Comparison of CerviHPV and Hybrid Capture 2 HPV tests for detection of high-risk HPV infection in cervical swab specimens. Diagn cytopathology. (2019) 47:439–44. doi: 10.1002/dc.24134, PMID: 30569591

[20] Li M Du X Lu M Zhang W Sun Z Li L. Prevalence characteristics of single and multiple HPV infections in women with cervical cancer and precancerous lesions in Beijing, China. J Med virology. (2019) 91:473–81. doi: 10.1002/jmv.25331, PMID: 30281807

[21] Wu C Zhu X Kang Y Cao Y Lu P Zhou W. Epidemiology of Humanpapilloma virus infection among women in Fujian, China. BMC Public Health. (2017) 18:95. doi: 10.1186/s12889-017-4651-7, PMID: 28774274 PMC5543557

[22] Vargas-Hernández VM Vargas-Aguilar VM Tovar-Rodríguez JM. Primary cervical cancer screening. Cirugia y cirujanos. (2015) 83:448–53. doi: 10.1016/j.circir.2014.09.001, PMID: 26162490

[23] Chatzistamatiou K Moysiadis T Angelis E Kaufmann A Skenderi A Jansen-Duerr P. Diagnostic accuracy of high-risk HPV DNA genotyping for primary cervical cancer screening and triage of HPV-positive women, compared to cytology: preliminary results of the PIPAVIR study. Arch gynecology obstetrics. (2017) 295:1247–57. doi: 10.1007/s00404-017-4324-x, PMID: 28337594

[24] Szarewski A Mesher D Cadman L Austin J Ashdown-Barr L Ho L. Comparison of seven tests for high-grade cervical intraepithelial neoplasia in women with abnormal smears: the Predictors 2 study. J Clin Microbiol. (2012) 50:1867–73. doi: 10.1128/jcm.00181-12, PMID: 22422852 PMC3372127

[25] Nakamura M Nakade K Orisaka S Iwadare J Mizumoto Y Fujiwara H. Comparison study of BD onclarity HPV with digene HC2 high-risk HPV DNA test and roche cobas 4800 HPV for detecting high-risk human papillomavirus in Japan. Am J Clin pathology. (2019) 151:263–9. doi: 10.1093/ajcp/aqy124, PMID: 30260388

[26] Chung HS Hahm C Lee M. Comparison of the clinical performances of the AdvanSure HPV Screening Real-Time PCR, the Abbott Real-Time High-Risk HPV Test, and the Hybrid Capture High-Risk HPV DNA Test for Cervical Cancer Screening. J virological Methods. (2014) 205:57–60. doi: 10.1016/j.jviromet.2014.04.021, PMID: 24814874

[27] Burroni E Sani C Bisanzi S Ocello C. HPV primary test in the cervical cancer screening: reproducibility assessment and investigation on cytological outcome of Hybrid Capture 2 borderline samples. Epidemiologia e prevenzione. (2016) 40:164–70. doi: 10.19191/ep16.3-4.Ad03.077, PMID: 27436249

[28] Cook DA Mei W Smith LW van Niekerk DJ Ceballos K Franco EL. Comparison of the Roche cobas^®^ 4800 and Digene Hybrid Capture^®^ 2 HPV tests for primary cervical cancer screening in the HPV FOCAL trial. BMC cancer. (2015) 15:968. doi: 10.1186/s12885-015-1959-5, PMID: 26674353 PMC4682219

