# Supplementary material for: Comparison of the SureX® HPV genotyping test with the Digene Hybrid Capture® 2 test in cervical cancer screening
Source: Front Oncol. 2025 Jul 24;15:1627935. doi: 10.3389/fonc.2025.1627935 (PMC12328181; doi:10.3389/fonc.2025.1627935)
Supplement: Supplementary file 1 [file Table1.docx]

Supplementary Material

# Supplementary Tables

**Supplementary Table 1** Distribution of positive results among 25 HPV genotypes detected by the SureX^®^ HPV test

| HPV types | Positive number | Positive rate (%) |
| --- | --- | --- |
| HPV53 | 95 | 3.14 |
| HPV52 | 88 | 2.91 |
| HPV16 | 75 | 2.48 |
| HPV58 | 71 | 2.34 |
| HPV42 | 69 | 2.28 |
| HPV51 | 56 | 1.85 |
| HPV81 | 55 | 1.82 |
| HPV43 | 48 | 1.59 |
| HPV56 | 45 | 1.49 |
| HPV31 | 43 | 1.42 |
| HPV33 | 42 | 1.39 |
| HPV68 | 39 | 1.29 |
| HPV39 | 35 | 1.16 |
| HPV66 | 30 | 0.99 |
| HPV18 | 24 | 0.79 |
| HPV6 | 20 | 0.66 |
| HPV59 | 16 | 0.53 |
| HPV44 | 13 | 0.43 |
| HPV83 | 13 | 0.43 |
| HPV45 | 9 | 0.30 |
| HPV82 | 9 | 0.30 |
| HPV11 | 5 | 0.17 |
| HPV26 | 5 | 0.17 |
| HPV35 | 3 | 0.10 |
| HPV73 | 3 | 0.10 |
